# Supplementary material for: Learning curve and surgical outcome of robotic assisted colorectal surgery with ERAS program
Source: Sci Rep. 2022 Nov 29;12:20566. doi: 10.1038/s41598-022-24665-w (PMC9709162; doi:10.1038/s41598-022-24665-w)
Supplement: Supplementary file 1 — Supplementary Information 1. [file 41598_2022_24665_MOESM1_ESM.zip › LSC Raw data-Ñ[▒K/065/H_ñw╝╨░O▒Kñσ.pdf]

正式摘要(電子簽章：000000185808698)<br>W75 / W75 ☐ 英文標題(注意:無英文姓名-請補病人基本檔!)	☐【讀取】憑證	

臺中榮民總醫院
出院病歷摘要

轉入號 : 轉出號 :

[redacted]

(8)流水編號	
(9)入院日期	109/02/05 (2020) 直腸外科 W75 - 012
(10)轉科日期	
(11)出院日期	109/02/14 (2020) 住院天數計 9 日

診斷

- 入院:Ca of Sigmoid colon, T1
- 出院:Ca of Sigmoid colon, T1 s/p Laparoscopic anterior resection and anastomosis on 2020/02/06

主訴

- incidentaly finding of sigmoid colon cancer, T1

病史

- This is a 46 year old male patient denied any systemic disease.

  According to the patient, he went to health examination and received colonoscopy which revealed Sigmoid colon T1 lesion which confirmed of adenocarcinoma. Therefore, he came to our CRS OPD for 2nd opinion. After discussion, the patient was admitted to our ward for surgical intervention.

體檢發現

- Anal inspection:
  Recto digital examination:
  Sigmoidoscopy:
  Anoscopy:
  Abdomen:
  - General appearance :
    - easy looking , conscious clear , without cardiopulmonary distress
    - Vital signs自動帶入date 體溫(BT) 脈搏(P) 呼吸(R) 血壓(BP) 身高(BH) 體重(BW) BMI
      20200205 36.3℃ 79次/分 16次/分 129/94mmHg 173.1cm 95.7kg 31.9
  - HEENT :
    - Eyes: no anemic conjunctiva, no icteric sclera, normal EOM
    - ENT: no otorrhea, no rhinorrhea, no erythematous mucosa, no injected tonsil, no deformity
  - Neck: supple, freely movable, trachea midline,no deformity, no engorged jugular vein , no palpable thyroid or lymph node enlargement
  - Chest:
    - Inspection: Grossly normal, symmetric and fully expansion, no accessory muscle used, no operation scar, no spider angiomata, no gynecomastia
    - Palpation: no palpable mass or nodule, no subcutaneous emphysema, no tenderness
    - Ausculation: clear breathing sound
  - Heart:
    - Percussion: normal heart size
    - Ausculation: regular heart beat, normal S1, S2, no gallop, no murmur
  - Abdomen:
    - Inspection: in
      - flat contour, symetrically, no scar, no stria, no dilated veins, no rashes
    - Ausculation: normoactive bowel sound, no abdominal bruits

-
  - palpation: soft, no muscle guarding or rigidity, no local tenderness, no rebounding pain, no palpable mass, no palpable pulsation
  - Percussion: no shifting dulllness, no hepatomegaly, no splenomagaly
-

Peripheral pulsation :	CA	BA	RA	FA	PA	PTA	DPA
Right side :	++	++	++	++	++	++	++/+++
Left side :	++	++	++	++	++	++	++/+++

- Genito-anus: no tenderness or palpable mass lesion or rectal shelf, normal external genitalia, normal anal tone, brown stool

CRS CONDITION

-
  - Lung

手術日期及方法

- 手術日期及方法20200206 1. Laparoscopic anterior resection
  2. Anastomosis: double stapling, EndoGIA 60-4.1mm & EEA 33mm
  3. Proximal cut end: 30 cm, distal cut end: 3.5cm
  4. Vessel ligation: IMA above left colic artery
  5. Take down the splenic flexure : yes.
  6.

住院治療經過

- after the admission, the patient recieved surgery on 2020/02/06 and tolerated it well. he recovered smoothly and was later discharged on 2020/02/14.

合併症

- nil

一般檢查

- 累積報告 :

DATE	TIME	ALB	TP	BIL.T	BIL.D	ALKP	AST	ALT	LDH	CHOL	TG	HDL-C	LDL	UA	NH3	AMY
1090205	1826	4.6	6.8	1.7	0.3	84	20	32	173							
1090206	1844			1.2	0.3			23	167							
1090210	0725			0.6	0.2		9	9								
1090213	0944	3.7		0.55	0.18		47	24								

DATE	TIME	WBC	RBC	HGB	HCT	MCV	PLT	BLST	NEUT	BAND	SEG	LYM	MON	EOS	BAS
1090205	1825	5730	5.37	16.7	47.9	89.2	168		60.0			31.8	5.8	1.9	0.5
1090206	1839	14390		15.8	46.4		143		83.8			11.9	3.8	0.3	0.2
1090210	0724	7540	5.18	16.3	46.6	90.0	165		71.0			19.9	5.6	3.2	0.3
1090213	0901	5680	4.96	15.4	44.7	90.1	190		60.2			27.8	5.8	5.5	0.7

DATE	TIME	NA	K	CL	CA	BUN	CREAT	Mg	P	CRP	ESR	PCT	LIPASE	LACTATE
1090205	1826	141	3.9	104	8.9	13	1.25							
1090206	1844	141	3.8		7.9	16	1.43							
1090207	0905				7.7									
1090210	0725	138	3.6	102	7.8	17	1.03							
1090210	0730													5.5
1090213	0804													7.6
1090213	0944	141	3.8			12	1.09							

DATE	TIME	GLUCOSE	PAB	TRF	ZN	CU	KETONE	CK	CKMB	PT-P	PT-C	APTT-P	APTT-C	TRO-T
1090205	1825									10.9	10.1	31.0		

- 放射線報告 :檢驗項目 **:CHEST PA VIEW** 報告日期 **:1090217**
  主　訴：
  Result:
  Finding:
  > tortuosity of thoracic aorta
  > no cardiomegaly
  > Engorgement of bilateral pulmonary hila.
  > increased interstitial infiltration of bilateral lower lung.
  Impression:
  As above description.

特殊檢查

病理報告

其它

出院時情況

- 3.改門診治療

出院指示

-

藥名	劑量	劑量單位	用法	頻次	數量	數量單位	現況	天數	單位
Voren-K tab 25mg	1	TAB	PO	TID	9	TAB	TH	3	天
Lactam tab 500mg	1	TAB	PO	Q6HPRN	12	TAB	TH	3	天
Ulstop tab 20mg	1	TAB	PO	BID	6	TAB	TH	3	天

出院計劃

主治醫師 2062F 蔣鋒帆 / 住院醫師 2328K 邱俊凱

正式摘要**(電子簽章：000000185808698)**

W75 / W75

doctorRank:B
